# Preeclampsia: The Interplay between Oxygen-Sensitive miRNAs and Erythropoietin

**DOI:** 10.3390/jcm9020574

**Published:** 2020-02-20

**Authors:** Vladislava Gusar, Angelika Timofeeva, Vitaliy Chagovets, Nataliya Kan, Oksana Vasilchenko, Kseniya Prozorovskaya, Tatyana Ivanets, Gennadiy Sukhikh

**Affiliations:** 1 Laboratory of Applied Transcriptomics, Federal State Budget Institution “National Medical Research Center for Obstetrics, Gynecology and Perinatology named after Academician V.I. Kulakov” of the Ministry of Healthcare of the Russian Federation, Oparin str. 4, Moscow 117997, Russia; v_timofeeva@oparina4.ru; 2 Laboratory of Proteomics and Metabolomics of Human Reproduction, Federal State Budget Institution “National Medical Research Center for Obstetrics, Gynecology and Perinatology named after Academician V.I. Kulakov” of the Ministry of Healthcare of the Russian Federation, Oparin str. 4, Moscow 117997, Russia; v_chagovets@oparina4.ru; 3 Department for Obstetrics and Gynecology, Professional Education Department, Federal State Budget Institution “National Medical Research Center for Obstetrics, Gynecology and Perinatology named after Academician V.I. Kulakov” of the Ministry of Healthcare of the Russian Federation, Oparin str. 4, Moscow 117997, Russia; n_kan@oparina4.ru; 4 Department of Innovative Technologies, Federal State Budget Institution “National Medical Research Center for Obstetrics, Gynecology and Perinatology named after Academician V.I. Kulakov” of the Ministry of Healthcare of the Russian Federation, Oparin str. 4, Moscow 117997, Russia; o_vasilchenko@oparina4.ru; 5 Obstetric Physiological Department, Federal State Budget Institution “National Medical Research Center for Obstetrics, Gynecology and Perinatology named after Academician V.I. Kulakov” of the Ministry of Healthcare of the Russian Federation, Oparin str. 4, Moscow 117997, Russia; k_prozorovskaya@oparina4.ru; 6 Clinical Diagnostic Laboratory, Federal State Budget Institution “National Medical Research Center for Obstetrics, Gynecology and Perinatology named after Academician V.I. Kulakov” of the Ministry of Healthcare of the Russian Federation, Oparin str. 4, Moscow 117997, Russia; t_ivanets@oparina4.ru; 7 Federal State Budget Institution “National Medical Research Center for Obstetrics, Gynecology and Perinatology named after Academician V.I. Kulakov” of the Ministry of Healthcare of the Russian Federation, Oparin str. 4, Moscow 117997, Russia; g_sukhikh@oparina4.ru

**Keywords:** microRNA/miRNA, preeclampsia, hypoxia/reoxygenation, erythropoietin, hemoglobin, deep sequencing, Real-Time qRT-PCR

## Abstract

Changes in the oxygen partial pressure caused by a violation of uteroplacental perfusion are considered a powerful inducer of a cascade of reactions leading to the clinical manifestation of preeclampsia (PE). At the same time, the induction of oxygen-dependent molecule expression, in particular, miRNA and erythropoietin, is modulated. Therefore, the focus of our study was aimed at estimating the miRNA expression profile of placental tissue and blood plasma in pregnant women with preeclampsia using deep sequencing and quantitative RT-PCR, as well as determining the concentration of erythropoietin. The expression of miR-27b-3p, miR-92b-3p, miR-125b-5p, miR-181a-5p, and miR-186-5p, as regulated by hypoxia/reoxygenation, was significantly increased in blood plasma during early-onset preeclampsia. The possibility of detecting early PE according to the logistic regression model (miR-92b-3p, miR-125b-5p, and miR-181a-5p (AUC = 0.91)) was evaluated. Furthermore, the erythropoietin level, which is regulated by miR-125b-5p, was significantly increased. According to PANTHER14.1, the participation of these miRNAs in the regulation of pathways, such as the hypoxia’s response via HIF activation, oxidative stress response, angiogenesis, and the VEGF signaling pathway, were determined.

## 1. Introduction

Preeclampsia (PE) is a complication of pregnancy with characteristic symptoms: arterial hypertension, proteinuria, and peripheral edema [1,2]. PE occurs in about 2–8% of pregnancies and clinically manifests after 20 weeks of gestation. PE is diagnosed based on clinical signs, Doppler sonography of the uteroplacental blood flow, and markers of placental dysfunction [2,3]. Methods of PE treatment are symptomatic. For severe PE, the only correct tactic is urgent delivery to avoid an increase in polyorganic failure in pregnant women.

The leading hypotheses based on the multifactorial pathogenesis of PE associate the disorder with impaired trophoblast invasion into the myometrial segments of the spiral arteries, which leads to a decrease in uteroplacental blood flow and irregular perfusion of the placenta [3,4,5]. As a result, hypoxia/reoxygenation occurs, leading to oxidative stress of the placenta—essentially in the endoplasmic reticulum compartment of the placental cells [6]. Stress induction promotes the release of pro-inflammatory cytokines, anti-angiogenic factors, and trophoblastic apoptotic debris, which cause activation of maternal endothelial cells and a subsequent generalized inflammatory response [7,8,9]. The cascade of processes launched by PE, in addition to the placenta, affects the cardiovascular system, liver, kidneys, and brain and activates the coagulation system [5]. It should be noted that these changes mainly characterize early-onset PE (<34 weeks of gestation). In late-onset PE (>34 weeks of gestation), diffuse placental hypoxia occurs for reasons not always associated with impaired placentation—in particular, due to overgrowing of the placenta, which leads to compression of the terminal villi, preventing perfusion [4,10].

The early development of the placenta occurs in an environment with low oxygen content through transcriptional and post-transcriptional regulation of angiogenic vascular growth factors, which are necessary for coordinating a successful pregnancy [7,11,12]. When placentation is impaired, a wide range of molecules that control gene expression are involved in the molecular mechanisms of the response to oxygen deprivation/reoxygenation, including small non-coding RNAs and microRNAs (miRNAs) [13,14]. MiRNAs are involved in key biological processes, such as proliferation, cell differentiation, and apoptosis [15,16], and their expression patterns are specific for various diseases [17]. Initially, miRNAs regulated by hypoxia were studied in tumors of various origins [18,19,20]. To date, more than 50 “hypoxamiRs” have been identified, among which miR-210 is assigned the leading role as an indicator of low oxygen tension in cells [13,14,21]. However, due to the use of different technological and experimental approaches, as well as the cellular specificity of “hypoxamiRs”, the existing data on their spectra vary [13]. The miRNA repertoire regulated by hypoxia/reoxygenation in various biological contexts remains uncertain, particularly in PE. It should be noted that changes in the partial pressure of oxygen contribute to the production of a number of other oxygen-dependent molecules, particularly erythropoietin (Epo). Contradictory data have been obtained on Epo’s significance in the pathogenesis of PE, despite the pleiotropic protective effects it exerts, including its effects on successful placentation and vascular adaptation during pregnancy, an increase in the duration of endothelial cell functioning during oxygen starvation, and increased proliferation and viability of the trophoblast [22,23,24,25,26,27]. Further, its expression is regulated by miR-125b-5p, which is a key participant in the HIF1α and VEGF signaling pathways [28].

In the context of the available data, we hypothesized that hypoxia/reoxygenation, due to impaired uteroplacental blood flow, activates oxygen-sensitive miRNAs. By regulating target genes, these miRNAs act as triggers for the signaling cascades associated not only with responses to hypoxia but also with the generalized inflammatory vascular reaction of the mother’s body. Based on this connection, our study focused on profiling miRNAs in the tissue and blood plasma of pregnant women with PE, with subsequent assessment of their expression by quantitative RT-PCR. The results obtained allowed us to determine miRNA’s participation in the regulation of signaling pathways, such as hypoxia’s response via HIF activation, oxidative stress response, and angiogenesis, as well as the VEGF signaling pathway, PDGF signaling pathway, and Endothelin signaling pathway. In addition, expression of miR-125b-5p and erythropoietin status in pregnant women with PE were evaluated. It should be noted that this study is a pilot, and this area has been understudied to the authors’ best knowledge.

## 2. Experimental Section

### 2.1. Patient Cohort

This study included pregnant women who were under observation at the “National Medical Research Center for Obstetrics, Gynecology, and Perinatology named after Academician V.I. Kulakov” of the Ministry of Healthcare of the Russian Federation. The total sample collection included 54 patients (cohort I) of reproductive age, from which two main groups of pregnant women were formed: those with early-onset PE (PE manifestation up to 34 weeks of gestation—*p* < 34; 16 pregnant women) and those with late-onset PE (PE manifestation after 34 weeks of gestation—*p* > 34, 12 pregnant). The group with early-onset PE included pregnant women with clinical signs of moderate (two women) and severe PE (14 women), and the group with late-onset PE comprised 11 pregnant women with moderate PE and one pregnant woman with severe PE. The control and main groups were matched by gestational age (10 pregnant women with preterm delivery after 34 weeks; 16 pregnant women with full-term physiological pregnancy). The clinical and biochemical characteristics of pregnant women included in the relevant groups are presented in Table 1.

To assess erythropoietin status and measure iron and nitrogen metabolism, 26 pregnant women (cohort II) with early-onset PE (12 pregnant women) and late-onset PE (14 pregnant women) were examined. The control group consisted of 18 pregnant women with normal pregnancies (Table 2).

The study did not include pregnant women who had vaginal delivery, multiple pregnancies resulting from IVF (in vitro fertilization), poor somatic anamnesis, chronic kidney disease, or the presence of genetic pathologies in the mother and fetus. The delivery of the pregnant women was performed by cesarean section. The estimation of fetal weight centiles is given in accordance with INTERGROWTH-21st (https://intergrowth21.tghn.org/translated-resources/). All studies were carried out with Patient Informed Consent in accordance with the Helsinki Declaration and were approved by the Commission of Biomedical Ethics at the “National Medical Research Center for Obstetrics, Gynecology, and Perinatology named after Academician V.I. Kulakov of the Ministry of Healthcare of the Russian Federation”.

### 2.2. RNA/miRNA Isolation from Placental Tissue and Peripheral Blood Plasma

Samples of placental tissue and peripheral blood plasma of the pregnant women, taken before and after the operation, were used for the experimental study.

Samples of a placenta tissue (a cross section through the maternal and fetal part of the placenta no more than 5 mm in thickness), taken immediately after delivery, were rinsed with a 0.9% solution of sodium chloride, placed in liquid nitrogen, and transferred to −75 °C for storage. The tissue was then homogenized in a QIAzol Lysis Reagent, and the total RNA was isolated by an miRNeasy MicroKit (Qiagen, Hilden, Germany) and enriched with a low molecular weight fraction of the miRNA with the RNeasy MinElute Cleanup Kit (Qiagen, Hilden, Germany). Quality control of the isolated samples with preliminary concentration measurements (Qubit 3.0, Invitrogen, Carlsbad, CA, USA) was carried out on an Agilent 2100 Bioanalyzer (Agilent Technologies, Santa Clara, CA, USA) using the RNA 6000 Nano Kit (Agilent Technologies). Total RNA samples with a 28S/18S ribosomal RNA ratio equal to 1.5–1.8 were stored at −75 °C for subsequent analysis.

Blood plasma was prepared according to the following protocol: whole blood was centrifuged at 300× *g*, 4 °C for 20 min, and then the supernatant was centrifuged at 14,000× *g* for 10 min. A total of 200 μL of the prepared plasma was used for the analysis, and 5.6 × 10^8^ copies of cel-miR-39 (miScript Primer Assay, Qiagen) were added to the plasma sample as an internal control for the efficiency of isolation and subsequent cDNA synthesis for quantitative RT-PCR. Then, miRNA was isolated by an miRNeasy Serum/Plasma kit (Qiagen). The extraction stages were carried out at an automatic station (QIAcube) in accordance with the protocols of the manufacturer Qiagen.

### 2.3. MiRNA Deep Sequencing

Evaluation of the miRNA expression profile in placental tissue and blood plasma was performed using high-throughput sequencing on a HiSeq 2000 platform (Illumina, San Diego, CA, USA). One microgram of total RNA from placental tissue or 250 ng of total RNA from blood plasma and a TruSeq Small RNA Sample Prep Kit (Illumina) were used to create cDNA libraries of the miRNA according to the manufacturer’s protocol. Quantitative and qualitative evaluation of the cDNA libraries was carried out using a High Sensitivity DNA chip and 2100 Bioanalyzer (Agilent Technologies). Sequencing data were processed with a HiSeq Reporter (Illumina). Reads with remote adapters (Novoalign, Novocraft Technologies Sdn Bhd, Selangor, Malaysia; http://www.novocraft.com/products/novoalign/ (parameters: -l 18 -h90 -r A) were aligned to the sequences from databases on the human genome Human Genome RefSeq Hg19 and miRBase 14.0 (http://www.mirbase.org). The number of reads was normalized according to the following formula: The number of copies of the detected miRNA divided by the total number of aligned reads, multiplied by 10^6^.

### 2.4. Real-Time Quantitative RT-PCR

The reverse transcription reaction was performed using an miScript II RT Kit (Qiagen). Quantitative PCR with the miScript SYBR Green PCR Kit (Qiagen) was performed using a StepOnePlus device (Applied Biosystems, Foster City, CA, USA) to determine the level of miRNA expression in the placental tissue and blood plasma. The following RNA-specific sense primers were used: hsa-miR-27b-3p MIMAT0000419 (5′-TTCACAGTGGCTAAGTTCTGC, Tm = 52 °C), hsa-miR- 92b-3p MIMAT0003218 (5′-TATTGCACTCGTCCCGGCCTCC, Tm = 52 °C), hsa-miR-125b-5p MIMAT0000423 (5′-TCCCTGAGACCCTAACTTGTGA, Tm = 59.5 °C), hsa-miR-181a-5p MIMA T0000256 (5′-AACATTCAACGCTGTCGGTGAGT, Tm = 56 °C) hsa-miR-186-5p MIMAT0000456 (5′-CAAAGAATTCTCCTTTTGGGCT, Tm = 52 °C), SNORD68 (5′-ACATTCTCCGGAATCGCTGT, Tm = 56 °C), cel-miR-39, and Tm = 55 °C. The stages were carried out in accordance with the Qiagen protocols. The threshold level of expression was Ct < 35. The level of miRNA expression was determined by the 2^−ΔΔCT^ method [29], using SNORD68 for placental tissue and a cel-miR-39 miScript Primer Assay (Qiagen) for blood plasma as the reference RNA.

### 2.5. Biochemical Measurement of Peripheral Blood in the Patient Cohort

The levels of endogenous erythropoietin (Epo) and peripheral blood parameters (iron, ferritin, transferrin, hemoglobin, hematocrit, urea, and creatinine) were determined in another group of pregnant women with PE. The study was carried out in accordance with the requirements of clinical and hematological examinations using the appropriate diagnostic test systems, the immunoassay Immulite 2000 (Siemens Healthcare Diagnostics Inc, Malvern, PA, USA), biochemical BA400 (BioSystems, Barcelona, Spain), and hematological Sysmex XS 800i/XT 2000i (Sysmex Corporation, Chuo-ku, Kobe, Hyogo, Japan) automated analyzers.

### 2.6. Statistical Data Analysis

The statistical significance of the difference between the biochemical parameters and the levels of miRNA expression in the groups under study was assessed by the Wilcox and Mann–Whitney methods using scripts written in the R language (https://www.R-project.org/). Logistic regression models for miRNA expression were created to test the possibility of using them as biomarkers. The efficiency of the created models was evaluated using ROC curves and the corresponding AUC values.

The normality of clinical parameters distribution was evaluated by the Shapiro-Wilk test. Statistical analysis was performed using the Student’s test with a normal distribution of the parameter and using the Mann-Whitney test when the distribution did not correspond to the law of normal distribution. To describe quantitative data having a normal distribution, the mean value (M) and standard deviation (SD) in the M ± SD format were used. In case of non-normal distribution, the parameter was described as the median (Me) and quartiles Q1, Q3 in the format Me (Q1–Q3).

## 3. Results

### 3.1. Profiling and Search for Hypoxia-Induced miRNAs in Placental Tissue and Blood Plasma at Early-Onset and Late-Onset PE

**Figure 1:**
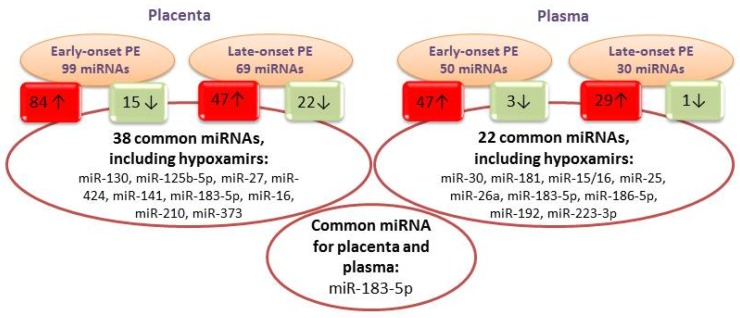
The spectrum of differentially expressed miRNAs in placental tissue and blood plasma in the early-onset and late-onset PE groups. The diagram indicates the number of miRNAs with altered expression; arrows indicate the direction of expression.

The miRNA high-throughput sequencing in single samples of placental tissue and peripheral blood plasma was conducted in pregnant women with early and late-onset PE, and in the control group. On average, the total number of aligned miRNA readings in the placental tissue samples in the PE groups and the control group was 6928382 million; in the blood plasma samples, it was 2848875 million. For the subsequent analysis, miRNAs with a minimum reading threshold of 100 and above and an expression fold change relative to the reference sample of >1.5 or <0.5 were selected. In the early-onset and late-onset PE groups, a wide range of differentially expressed miRNAs was determined (Figure 1).

Comparing the data on differential expression, we found 38 miRNAs with altered expression levels in placental tissue that were common to the two groups. A similar analysis performed on the blood plasma samples revealed 22 miRNAs, among which we discovered the miRNAs described previously as “hypoxamiRs” in a number of cell lines [13].

Only one miRNA common among the two groups (in placental tissue or plasma) was detected: miR-183-5p. Interestingly, the expression fold changes of these miRNAs in late-onset PE varied from 1.5–5.6 times, while in the early-onset PE, this change was 2.5–21 times. This change may be caused by the increased secretion of placental factors into the blood flow—likely because of hypoxia—and is associated with the severity of PE. The number of unique miRNAs found only in blood plasma for early-onset PE was 1,5 times greater than that for late-onset PE.

### 3.2. Evaluation of Hypoxia-Induced miRNA Expression

**Figure 2:**
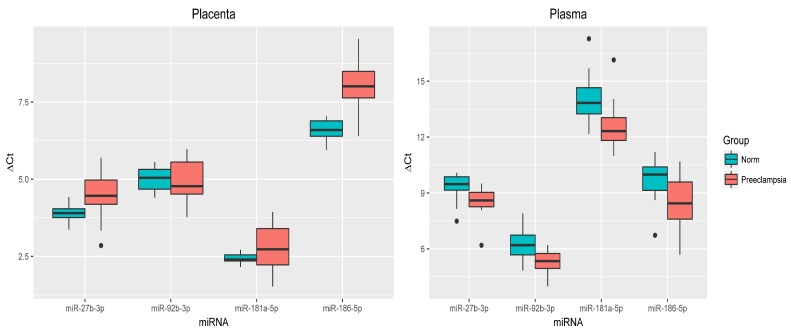
Comparative analysis of the expression levels for miR-27b-3p, miR-92b-3p, miR-181a-5p, and miR-186-5p in the placental tissue and blood plasma from pregnant women with early-onset PE. The box diagrams indicate the medians of the ΔCt values, the first and third quartiles, and the edges of the statistically significant samples, while the dots denote the emissions. The lower the value of ΔCt, the higher the level of expression of the studied miRNAs.

Based on the sequencing data, 36 miRNAs were selected, of which four miRNAs (miR-27b-3p, miR-92b-3p, miR-181a-5p, and miR-186-5p) were verified by quantitative RT-PCR. This verification was carried out in the blood plasma samples taken before surgery and in the placental tissue from the group of pregnant women with PE, as well as the control groups of the appropriate gestational age. An analysis of the differential miRNA expression showed that in the placental tissue of pregnant women with early-onset PE, the expression levels of miR-92b-3p and miR-181a-5p did not change (*p* > 0.05), while miR-27b-3p and miR-186-5p significantly decreased (*p* < 0.003) with respect to the control group. In contrast, the expression of miR-27b-3p (*p* < 0.03), miR-92b-3p (*p* < 0.01), miR-181a-5p (*p* < 0.006), and miR-186-5p (*p* < 0.02) was significantly higher in blood plasma (Figure 2).

**Figure 3:**
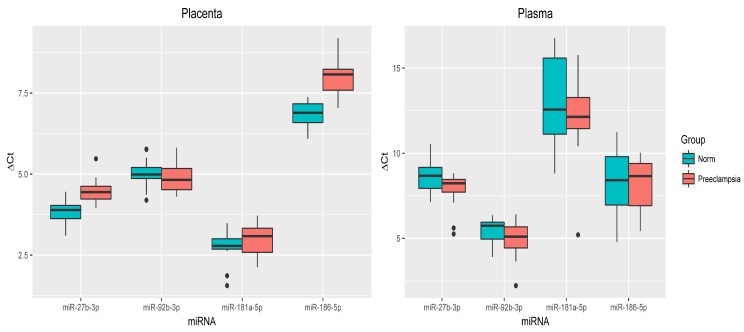
Comparative analysis of the expression levels for miR-27b-3p, miR-92b-3p, miR-181a-5p, and miR-186-5p in placental tissue and blood plasma samples from pregnant women with late-onset PE. The box diagram indicates the medians of the ΔCt values, the first and third quartiles, and the edges of the statistically significant samples, while the dots denote the emissions. The lower the value of ΔCt, the higher the level of expression of the studied miRNAs.

In pregnant women with late-onset PE, the above miRNAs did not show significant differences in blood plasma (*p* > 0.05). In the placental tissue, only miR-27b-3p and miR-186-5p expression (*p* < 0.001) was significantly decreased, as in the group with early-onset PE (Figure 3).

It should be noted that the directionality of changes in the expression levels of the studied miRNAs in the blood plasma from pregnant women with early-onset PE according to the sequencing data coincided with the data obtained by quantitative RT-PCR, except for miR-92b-3p, whose expression, according to the sequencing data, decreased. Most likely, this discrepancy is due to the miRNA verification of the extended sample.

### 3.3. Evaluation of Expression of miR-125b-5p Acting as an Erythropoietin Modulator in Placental Tissue and Blood Plasma

**Figure 4:**
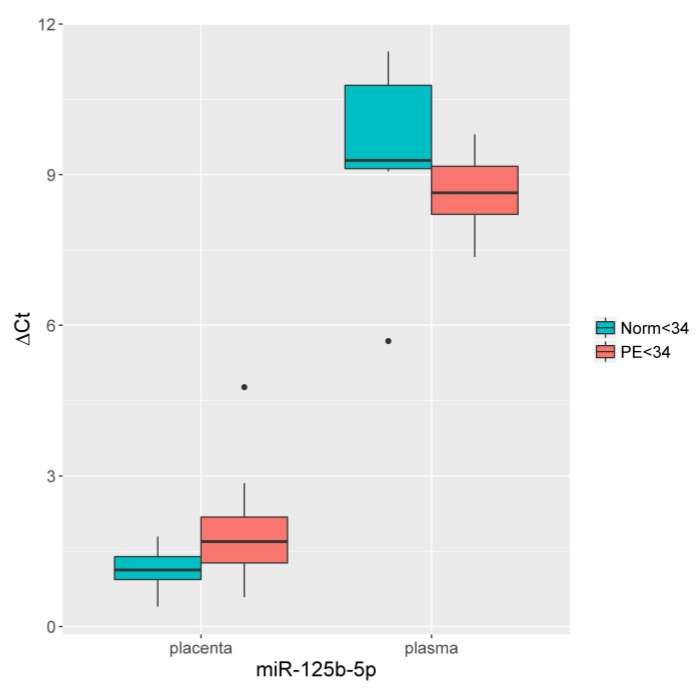
Comparative analysis of the miR-125b-5p expression level in the placental tissue and blood plasma from pregnant women with early-onset PE. The box diagram indicates the medians of the ΔCt values, the first and third quartiles, and the edges of the statistically significant samples, while the dots denote the emissions. The lower the value of ΔCt, the higher the level of expression of the studied miRNAs.

The miR-125b-5p expression was estimated by quantitative RT-PCR in the placental tissue and blood plasma, taken before delivery from pregnant women with early-onset PE and from the control groups with the relevant weeks of gestation. Significant changes in the miR-125b-5p expression level were detected: expression decreased in the placental tissue (*p* < 0.01) and increased in blood plasma *(p* < 0.04) relative to the control group, as shown in Figure 4.

### 3.4. Evaluation of Hypoxia-Induced miRNA Expression in Blood Plasma on the 1st Day After Delivery

**Figure 5:**
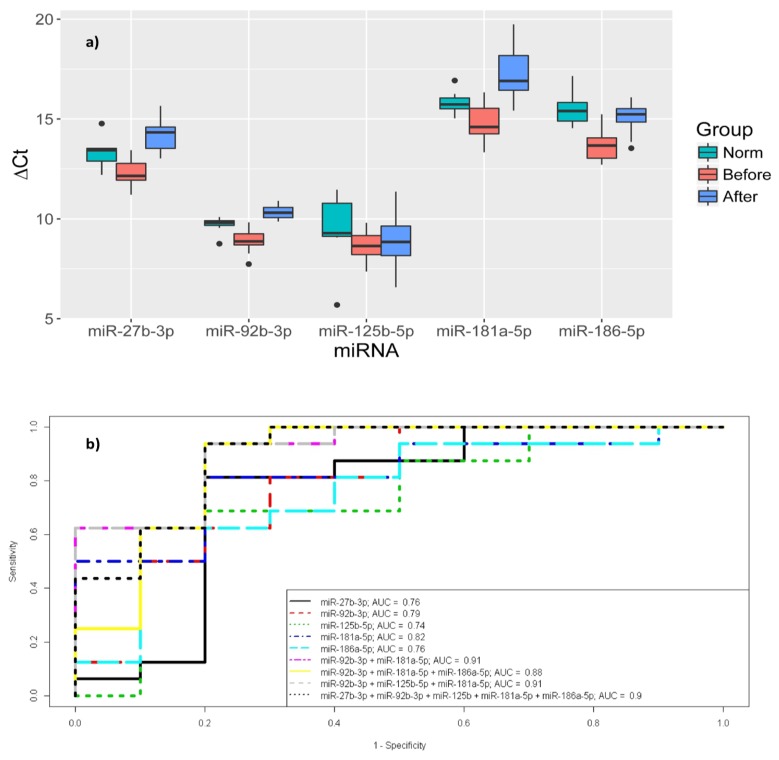
(**a**) Comparative analysis of miR-27b-3p, miR-92b-3p, miR-125b-5p, miR-181a-5p, and miR-186-5p expression levels in the blood plasma samples taken before (Before) and on the first day after delivery (After) from pregnant women with early-onset PE and from the control group before delivery (Norm). The box diagram indicates the medians of the ΔCt values, the first and third quartiles, and the edges of the statistically significant sample, while the dots denote the emissions. The lower the value of ΔCt, the higher the level of expression of the studied miRNAs. (**b**) The ROC-curves of the logistic regression models for miR-27b-3p, miR-92b-3p, miR-125b-5p, miR-181a-5p, and miR-186-5p expression levels in early-onset PE and in the control group with relevant weeks of gestation. The AUC values for each model are also indicated.

Considering the obtained data on the significant changes of miR-27b-3p, miR-92b-3p, miR-125b-5p, miR-181a-5p, and miR-186-5p expression in blood plasma before delivery in early-onset PE, we estimated their expression on the first day after delivery. This analysis was carried out by quantitative RT-PCR in the blood plasma samples taken from the same pregnant women (Figure 5a).

The expression levels of these miRNAs, except for miR-125b-5p, significantly decreased on the first day after delivery (*p* < 0.001), approximating normal values. For miR-125b-5p, there was a slight tendency to decrease expression, but no statistically significant changes were observed (*p* > 0.05). To assess the possibility of differentiation between patients with and without PE, the ROC-curves for the logistic regression models were constructed based on the expression levels of the studied miRNAs (Figure 5b). A model with high AUC values (0.91) indicates the possibility to detect early PE based on estimates of miRNA expression levels: miR-92b-3p + miR-181a-5p and miR-92b-3p + miR-125b-5p + miR-181a -5p. It is important to note that the AUC value was established as 0.9 for all five miRNAs: miR-27b-3p + miR-92b-3p + miR-125b-5p + miR-181a-5p + miR-186-5p.

### 3.5. Evaluation of Erythropoietin Status, Iron and Nitrogen Metabolism in Pregnant Women with PE

Hypoxia of various origins, including placental hypoxia, induces Epo production, the expression of which stimulates angiogenesis and has anti-apoptotic effects. The anemia and iron deficiency conditions observed during pregnancy can also contribute to the increased production of Epo. Considering this dependence, we evaluated Epo concentrations in the serum of pregnant women with early-onset and late PE relative to the control group. The values of hemoglobin, hematocrit, and iron metabolism (iron, ferritin, and transferrin) were also estimated. Furthermore, the values of urea and creatinine were studied since, in cases of PE, perfusion decreases in the kidneys such that relative hypoxemia leads to an increase in Epo production (Table 3).

The parameters of iron and nitrogen metabolism did not reveal significant differences between the three groups (*p* > 0.05). In the group with early-onset and late PE, the serum EPO concentration was significantly higher relative to the control group: 0.27 ± 0.08 mIU/mL (*p* < 0.05) and 0.43 ± 0.12 mIU/mL (*p* < 0.04), respectively, with a tendency to increase in late-onset PE. The levels of hemoglobin and hematocrit showed a slight decrease with respect to the control group. However, statistically significant differences were revealed for hematocrit (*p* < 0.04).

## 4. Discussion

Studies in search of the initiating factors of the cascade of processes observed in PE have made it possible to reach a wide range of regulatory molecules, such as miRNAs. Many works have presented specific patterns of miRNA expression in cultured trophoblasts [30,31,32], placental tissues [33,34,35,36,37,38], and circulating in the maternal blood flow as a result of trophoblast cell secretion during PE [39,40,41,42]. However, given the multifactorial nature of the pathogenesis of PE, it is assumed that a specific miRNA repertoire is involved in the finely tuned network that regulates each link in this cascade—in particular, the link associated with hypoxia/reoxygenation.

Thus, in order to evaluate the full spectrum of tissues and circulating miRNAs in pregnant women with early- and late-onset PE, deep sequencing was performed. A comparison of the expression profiles revealed a wide pattern of tissue miRNAs that are differentially expressed in both groups. Among them, trophoblast miRNAs (miR-515-517, -517c, -519a, and -527) were observed, and miRNAs were expressed in response to hypoxia (as shown earlier): let-7 (b / c / d / e), miR -16-5p, -27a-3p, -29a-3p, -125b-5p, -126-5p, -141-3p, -183-5p, -210-3p, -423-5p, and -424-5p [16,19,20,43]. The majority of the miRNAs exhibited increased expression. Only expression of the let-7 family, miR-130b-3p, and miR-423-5p was decreased. More than a twofold decrease in the expression levels of let-7c, miR-423-5p, -519a-3p, and a number of other miRNAs in the placenta was observed for PE in our previous studies [44,45]. The increased secretion of extracellular vesicles of trophoblastic origin containing miRNAs into the maternal blood flow due to placental dysfunction in PE can contribute to systemic biological changes. Among the miRNAs circulating in blood plasma, we identified (let-7g-5p, miR-26a-5p, miR-28 (-28-3p, -151a-3p), -30a-5p, -148a-3p, -181 (-181a-5p), -183-5p, -186-5p, -188 (-532-5p), -192-5p, and -320 (-320a), which change their expression in response to oxygen deprivation [18,19,20], oxidative stress (miR-140-3p, -143-3p, -378a-3p) [46], and are associated with immune and inflammatory responses (miR-375) [47], as well as ischemia/reperfusion (miR-423-3p) [48]. In general, our results are consistent with the data of other authors. The discrepancy in our results on the direction of expression changes of some miRNAs can possibly be explained through the use of different technological approaches and research platforms for studies, as well as taking biological materials and matching the gestational age in the compared groups.

The hypoxic effects observed during abnormal placentation are mainly mediated by the transcription factor induced by the hypoxia, HIF1 [49]. HIF1 can exert multilevel effects on the “hypoxamiRs” network by directly binding to the HRE (hypoxia regulatory elements) located in the promoter regions of a number of miRNAs [13,20]. However, a significant number of miRNAs under conditions of low oxygen availability can be regulated by HIF-independent pathways, including modulation of inflammatory responses [50] and activation of endothelial cells [13,51]. Notably, Truong et al. showed that trophoblast cells cultured under hypoxia secreted miRNAs associated with the inflammatory response and regulation of cytokine production [52].

Given the existing data, it seems interesting to us to identify miRNAs whose expressions change in response to placental hypoxia or reoxygenation. To that end, according to the sequencing data, we selected 36 miRNAs, and 4 miRNAs among them (miR-27b-3p, miR-92b-3p, miR-181a-5p, and miR-186-5p) were verified by quantitative RT-PCR in blood plasma taken before delivery and in the placental tissue samples from pregnant women with PE, corresponding to the control groups (54 samples in total). A comparative analysis showed a significant expression decrease for miR-27b-3p and miR-186-5p in the placental tissue in pregnant women with early and late-onset PE, whereas in the plasma, all four miRNAs (miR-27b-3p, miR-92b-3p, miR-181a-5p, and miR-186-5p) were significantly increased only in pregnant women with early-onset PE. Earlier, an increase in the expression of miR-181a-5p was demonstrated in placental tissue [36,38] and blood plasma in PE [40], and the differential expression of miR-92b-3p in the placenta was observed in the study of S.-Y.Choi et al. [53]. In a number of studies on experimental models, the expression of miR-27b-3p, miR-92b-3p, and miR-181a-5p was shown to change in response to ischemic damage to brain neurons [54,55]. We suggest that the altered expression of the studied miRNAs in placental tissue may indicate the adaptation of the cells to fluctuations in the partial pressure of oxygen, as well as in blood plasma—for their extracellular export, regulated by hypoxia or reoxygenation. In particular, the experiments of Hale et al. showed extracellular miR-210 to be a highly regulated effector in maintaining control of the hypoxic adaptation between anatomically different cells [56]. It is interesting to note the differences in the directivity of miRNA expression levels in the placental tissue and plasma of pregnant women. There is evidence that the miRNA expression patterns of the original cell and those secreted by the exosomes are different [57]. Moreover, an extra-placental source of plasma miRNAs is not excluded. We also focused on the fact that in late-onset PE, none of the miRNAs showed altered expression in blood plasma. Given the differences in the pathogenesis of early- and late-onset PE, it is suggested that the induction of the expression of these miRNAs may be due to placental hypoxia/reoxygenation, which is characteristic of early-onset PE.

The existing data on the involvement of miR-125b-5p in immune and inflammatory responses, oxidative stress, hypoxia [58], and the regulation of Epo expression [28] determined its choice for inclusion our study. The expression of miR-125b-5p showed a significant decrease in the placental tissue and an increase in plasma from the pregnant women with early-onset PE. Previously, a decrease in miR-125b-5p expression was demonstrated in pregnant women with early fetal growth retardation [33], PE, and arterial hypertension [59]. Moreover, the participation of miR-125b-5p expression in the pathogenesis of PE was confirmed [60]. We suggest that the increased expression of miR-125b-5p, as well as that of miR-27b-3p, miR-92b-3p, miR-181a-5p, and miR-186-5p, in blood plasma is the result of the local effects of placental hypoxia/reoxygenation mediated by the transcription of HIF1α and its targets. In response to changes in oxygen concentration, HIF1, as the main regulator of adaptive metabolic reactions of the body to hypoxia, activates the transcription of the target genes associated with improved oxygen delivery to tissues, such as VEGF and Epo [43].

It is known that the main function of Epo is the regulation of erythropoiesis under hypoxia [61]. In addition, Epo promotes successful placentation and vascular adaptation during pregnancy [62]. There are few studies on its role in changes associated with placental dysfunction [22,23,24,25,62,63]. The authors indicate an increase in the concentration of Epo in the placenta [23], plasma [24,25], and the serum of pregnant women with PE [22], as well as a correlation of its level with the acid–base parameters in umbilical cord blood and neonatal outcomes [63]. However, the importance of Epo in the pathogenesis of PE, as before, remains controversial. Given its significant role in the formation of protective effects of the body in response to hypoxia, we evaluated the erythropoietin status in the pregnant women with PE and in the control group (44 samples in total).

A significant increase in Epo was found in the group with early and late PE, with a tendency to increase its level in late PE. Epo levels increased during pregnancy by 2–4 times, reaching a plateau level after 20 weeks, likely due to physiological hemodilution [24,26,27]. It is also known that anemia during pregnancy leads to increased production of Epo in response to a decrease in hemoglobin and iron deficiency [26,27]; further, an increase in blood volume and a decrease in viscosity contribute to good fetal oxygenation during pregnancy [26]. In our work, the hemoglobin and hematocrit values were slightly reduced. However, significant differences were observed for hematocrit. The decrease in serum iron, ferritin, and transferrin levels in PE was not statistically significant. Assuming an increase in Epo is associated with a decrease in renal perfusion, the nitrogen metabolism indices (creatinine and urea) were determined not to differ significantly, which may support the extrarenal secretion of Epo and is consistent with the data of Hershkovitz et al. [24]. The results suggest that an increase in the level of Epo in pregnant women with PE is due to its expression in response to changes in the oxygen concentration in the placenta. This is also supported by our data on the decrease in the expression of miR-125b-5p, the target of which is Epo, in placental tissue from pregnant women with PE. This relationship has been confirmed by Ferracin et al. to occur through the transfection of breast cancer cell lines, where the reduced expression of miR-125b-5p in metastatic breast cancer induced the overexpression of Epo and its receptor [28]. However, similar studies using Luciferase reporter assays on placental tissues of pregnant women with PE have not been performed.

Considering the obtained data, it seemed interesting to us to assess the possibility of differentiating between patients with and without PE based on their expression levels of the studied miRNAs. To accomplish this, a logistic regression model with a high AUC value was constructed, which indicates that these miRNAs allow the detection of early PE (miR-92b-3p + miR-125b-5p + miR-181a-5p (AUC = 0.91). However, to confirm the use of these miRNAs as diagnostic markers, their verification in the blood plasma of pregnant women from the risk group for the development of PE in the early stages of gestation is necessary.

**Figure 6:**
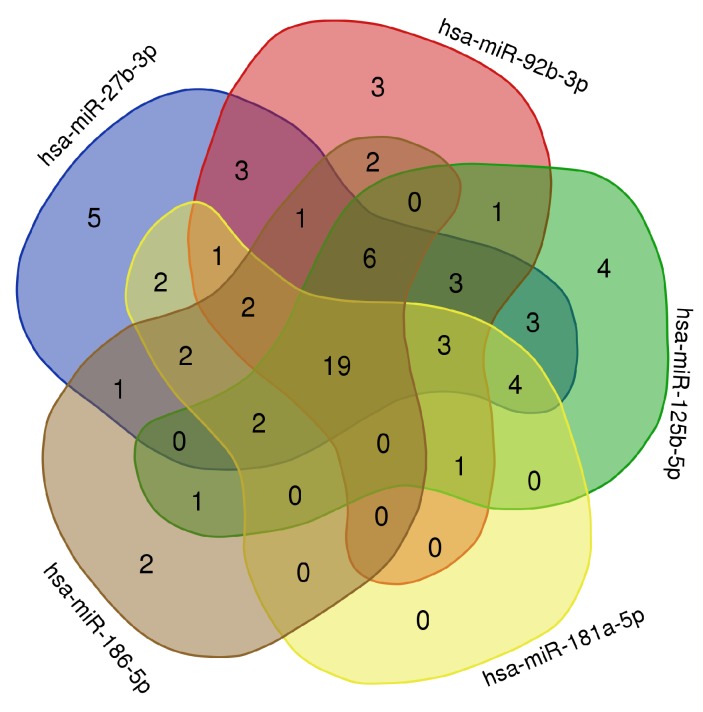
Venn Diagram. This diagram indicates the number of signaling pathways (*р*-value ≤ 0.05) whose potential regulation is possibly affected by the studied miRNAs.

Our analysis of the PANTHER14.1 database revealed a number of pathways that are common for all five miRNAs and characteristic for their specific combinations (Figure 6, Table 4).

Ultimately, all five studied miRNAs are involved in the regulation of a number of signaling pathways that are associated with normal placental development. Moreover, their individual combinations are involved in the activation of cascades that are associated, in particular, with responses to hypoxia, oxidative stress, and the VEGF signaling pathway. The data obtained by Bertero et al. indicate that one miRNA can subordinately control other “hypoxamiRs” and target gene networks, thereby regulating the signaling pathways in various cellular contexts [64].

**Figure 7:**
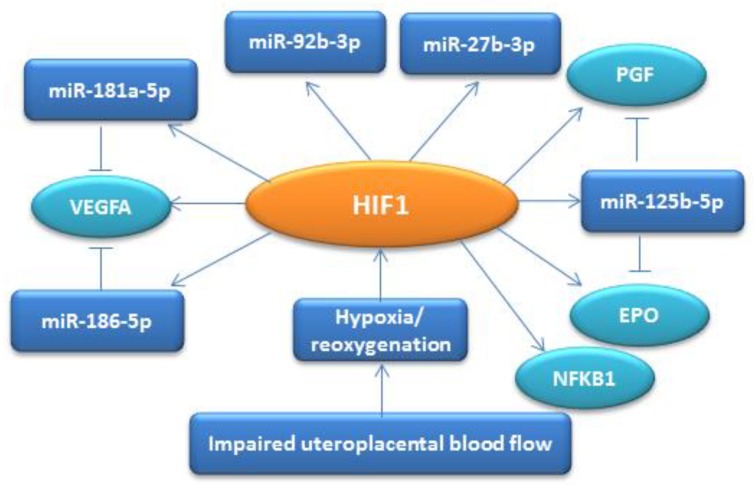
Schematic model showing the activation of a system regulated by HIF1 induced by hypoxia/reoxygenation. VEGF is the vascular growth factor, PGF is the placental growth factor, EPO is erythropoietin, and NFKB1 is a nuclear transcription factor, the activation of which is associated with inflammatory reactions. Arrows indicate the induction of miRNA and gene expression. Lines in the form of the letter “T” indicate the target genes for miR-181a-5p, miR-186-5p, and miR-125b-5p.

It is also known that miRNAs under the conditions of oxygen deprivation can be regulated by HIF-independent transcription factors associated with inflammation, particularly NF-κB (nuclear factor-kappaB) [50]. Thus, in the context of existing data and the results of our study, we propose a model wherein, due to impaired uteroplacental blood flow, the resulting hypoxia or reoxygenation induces the expression of miR-27b-3p, miR-92b-3p, miR-125b-5p, miR-181a -5p, and miR-186-5p, as well as Epo. This induction is mediated by the activation of a system regulated by HIF1, in which various genes and signaling cascades are involved, including those associated with inflammation and vascular dysfunction. In response to fluctuations in oxygen concentration, this system can exert modulating adaptive protective effects, which are supported by an increase in plasma Epo in pregnant women with PE (Figure 7).

## 5. Conclusions

In conclusion, this study showed that in response to changes in the partial pressure of oxygen caused by a violation of uteroplacental perfusion, oxygen-dependent miRNAs and Epo are activated. These miRNAs and Epo are involved in a finely tuned system that regulates one of the links in the pathogenesis of PE associated with hypoxia/reoxygenation. However, to better understand the molecular mechanisms underlying the pathophysiological reactions to oxygen deprivation/reoxygenation in PE, further studies that focus on the participation of “hypoxamiRs” in specific signaling cascades are needed.

## Figures and Tables

**Table 1:** The clinical and biochemical characteristics of pregnant women (cohort I).

	<34 GW			>34 GW		
	Pregnant Women Cohort with PE (*n* = 16)	Control Group (*n* = 10)	*p*-Value	Pregnant Women Cohort with PE (*n* = 12)	Control Group (*n* = 16)	*p*-Value
Gestational age at the time of delivery, weeks	31 ± 3	30 ± 3	0.42	37 ± 2	37 ± 3	1
Manifestation PE, weeks	28 ± 4	absent	-	36.5 ± 1	absent	-
Systolic blood pressure (110–130 mmHg)	153.6 ± 18.3	113.4 ± 4.8	<0.001	142.08 ± 14.2	112.4 ± 4.7	<0.001
Diastolic blood pressure (65–80 mmHg)	98.6 ± 11.1	68.2 ± 3.8	<0.001	91.6 ± 9.3	68.3 ± 3.4	<0.001
Proteinuria (0–0.2 g/L)	3.53 (1.28–4.37)	absent	-	0.7 (0.17–2.92)	absent	-
Peripheral edema, *n* (%)	7 (43.7%)	absent	-	8 (66.7%)	absent	-
Ratio of placental dysfunction markers (sFLT-1/PLGF; 1.5–7)	315.65 (134.28–475.18)	NA	-	173.3 ± 116.04	NA	-
Platelet level (150–400 × 10^9^ c/L)	204 ± 77.04	238.5 ± 54.9	0.2	212.08 ± 56.63	227.6 ± 61.5	0.5
Liver function test:						
ALT level (0–40 u/L)	23.05 (11.8-59.32)	NA	-	20.8±7.6	NA	-
AST level (0–40 u/L)	27.15 (17.08-35.53)	NA	-	25.4±9.2	NA	-
Birth weight, grams (centiles)	1587 ± 204 (42.53 ± 29.82)	VLBW -	-	2912 ± 168 (55.55 ± 32.09)	3457 ± 133 (75.92 ± 30.04)	<0.001

NA–Not analysed. GW–gestational week. VLBW–very low birth weight. For normal distribution, the mean value (M) and standard deviation (SD) in the M ± SD format were used. In case of non-normal distribution-the median (Me) and quartiles Q1, Q3 in the format Me (Q1–Q3) were used.

**Table 2:** The clinical and biochemical characteristics of pregnant women (cohort II).

	Pregnant Women Cohort with PE		*p*-Value <34 GW vs. >34 GW	Control Group (*n* = 18)	*p*-Value	
	<34 GW (*n* = 12)	>34 GW (*n* = 14)			<34 GW	>34 GW
Gestational age at the time of delivery, weeks	30 ± 2	37 ± 1	<0.001	39 ± 1	<0.001	<0.001
Systolic blood pressure (110–130 mmHg)	147.1 ± 19.9	138.0 ± 17.3	0.245	114.3 ± 6.0	<0.001	<0.001
Diastolic blood pressure (65–80 mmHg)	94.5 ± 14.4	86.1 ± 13.8	0.156	72.1 ± 5.9	<0.001	0.002
Proteinuria (0–0.2 g/L)	0.65 (0.12–1.44)	1.07 (0.41–4.17)	0.265	absent	-	-
Peripheral edema, *n* (%)	6 (50.0%)	7 (50.0%)	-	absent	-	-
Ratio of placental dysfunction markers (sFLT-1/PLGF; 1.5–7)	261.0 ± 96.5	175.10 (109.18–324.45)	0.549	NA	-	-
Platelet level (150–400 × 10^9^ c/L)	237.5 ± 71.3	237.00 (207.75–256.00)	0.537	237.5 ± 43.2	1	0.94
Liver function test:						
ALT level (0–40 u/L)	29.75 (18.33–34.90)	62.4 ± 31.1	0.225	NA	-	-
AST level (0–40 u/L)	25.6 (19.68–32.90)	53.2 ± 23.4	0.852	NA	-	-
Birth weight, grams (centiles)	1689 ± 831 (40 ± 27.39)	2939 ± 575 (62.86 ± 26.71)	0.001	3531 ± 134 (82.33 ± 25.27)	<0.001	0.001

NA–Not analysed. GW–gestational week. For normal distribution, the mean value (M) and standard deviation (SD) in the M ± SD format were used. In case of non-normal distribution - the median (Me) and quartiles Q1, Q3 in the format Me (Q1–Q3) were used.

**Table 3:** Measurement of erythropoiesis, iron metabolism, and nitrogen metabolism in the cohort of pregnant women with PE and in the control group.

	Pregnant Women Cohort with PE		Control Group (*n* = 18)	*P*-Value	
	<34 GW (*n* = 12)	>34 GW (*n* = 14)		<34 GW	>34 GW
Erythropoietin, МЕ/mL *	0.27 ± 0.08	0.43 ± 0.12	0.05 ± 0.01	0.05	0.04
Haemoglobin, g/L	114.2 ± 32.3	109.3 ± 29.2	121.1 ± 28.5	0.27	0.07
Haematocrit, l/L *	0.34 ± 0.10	0.34 ± 0.09	0.37 ± 0.09	0.04	0.04
Ferritin, μg/L	23.09 ± 6.6	19.10 ± 5.1	25.08 ± 5.9	0.15	0.47
Transferrin, mg/dL	420.04 ± 121.2	494.1 ± 132	432.7 ± 102	0.24	0.79
Iron, µM	17.2 ± 4.9	13.8 ± 3.7	18.6 ± 4.4	0.07	0.06
Blood urea, mmol/L	3.08 ± 0.9	4.07 ± 1.09	2.9 ± 0.7	0.78	0.06
Creatinine, mmol/L	70.3 ± 20.3	77.9 ± 20.8	73.4 ± 17.3	0.65	0.06

The relative units normalized to the range of the reference values of erythropoietin are indicated. GW—gestational week. M ± SD. * *p*-value < 0.05.

**Table 4:** Combinations of the studied miRNAs that potentially regulate the signaling pathways associated with normal placental development and its dysfunction.

Combinations of miRNAs	Signaling Pathway Potentially Regulated by Studied miRNAs (*p*-Value ≤ 0.05)
hsa-miR-27b-3p/hsa-miR-92b-3p/hsa-miR-125b-5p/hsa-miR-181a-5p/hsa-miR-186-5p	Angiogenesis FGF signaling pathway
	PDGF signaling pathway
	EGF receptor signaling pathway
	TGF beta signaling pathway
	Endothelin signaling pathway
	Apoptosis signaling pathway
	Interleukin signaling pathway
	Wnt signaling pathway
	Oxytocin receptor mediated signaling pathway
hsa-miR-27b-3p/hsa-miR-92b-3p/hsa-miR-125b-5p/hsa-miR-181a-5p	VEGF signaling pathway PI3 kinase pathway
hsa-miR-27b-3p/hsa-miR-92b-3p/hsa-miR-186-5p	Hypoxia response via HIF activation
hsa-miR-27b-3p/hsa-miR-92b-3p/hsa-miR-125b-5p/hsa-miR-186-5p	Oxidative stress response Hedgehog signaling pathway p53 pathway by glucose deprivation
hsa-miR-27b-3p/hsa-miR-125b-5p/hsa-miR-181a-5p	Inflammation mediated by chemokine and cytokine signaling pathway
hsa-miR-27b-3p/hsa-miR-181a-5p/hsa-miR-186-5p	Ubiquitin proteasome pathway
	Cadherin signaling pathway
